# Characterization of a Fungal Virus Representing a Novel Genus in the Family *Alphaflexiviridae*

**DOI:** 10.3390/v15020339

**Published:** 2023-01-25

**Authors:** Ting Ye, Zhongbo Lu, Han Li, Jie Duan, Du Hai, Yang Lin, Jiatao Xie, Jiasen Cheng, Bo Li, Tao Chen, Yanping Fu, Daohong Jiang

**Affiliations:** 1 State Key Laboratory of Agricultural Microbiology, Huazhong Agricultural University, Wuhan 430070, China; 2 Hubei Key Laboratory of Plant Pathology, College of Plant Science and Technology, Huazhong Agricultural University, Wuhan 430070, China

**Keywords:** flexivirus, mycovirus, *Sclerotinia sclerotiorum*, Alphaflexiviridae

## Abstract

*Sclerotinia sclerotiorum* is an ascomycetous fungus and hosts various mycoviruses. In this study, a novel fungal alphaflexivirus with a special genomic structure, named *Sclerotinia sclerotiorum* alphaflexivirus 1 (SsAFV1), was cloned from a hypovirulent strain, AHS31. Strain AHS31 was also co-infected with two botourmiaviruses and two mitoviruses. The complete genome of SsAFV1 comprised 6939 bases with four open reading frames (ORFs), a conserved 5′-untranslated region (UTR), and a poly(A) tail in the 3′ terminal; the ORF1 and ORF3 encoded a replicase and a coat protein (CP), respectively, while the function of the proteins encoded by ORF2 and ORF4 was unknown. The virion of SsAFV1 was flexuous filamentous 480–510 nm in length and 9–10 nm in diameter. The results of the alignment and the phylogenetic analysis showed that SsAFV1 is related to allexivirus and botrexvirus, such as Garlic virus X of the genus *Allexivirus* and Botrytis virus X of the genus *Botrevirus,* both with 44% amino-acid (aa) identity of replicase. Thus, SsAFV1 is a novel virus and a new genus, Sclerotexvirus, is proposed to accommodate this novel alphaflexivirus.

## 1. Introduction

*Sclerotinia sclerotiorum* (Lib.) de Bary, a destructive ascomycetous fungus, infects more than 700 plant species, some of which are economically important crops, such as *Brassica napus* (rapeseed), *Helianthus annuus* L. (sunflower), *Arachis hypogaea* (peanut), *Glycine max* (soybean), *Beta vulgaris* (sugar beet), and *Lactuca sativa* (garden lettuce) [1,2,3,4]. This fungus has evolved complicated pathogenicity mechanisms, including hydrolases, oxalic acid, secreted proteins, and other pathogenic proteins, to attack and destroy host tissue in a short period [5,6]. The fungus *S. sclerotiorum* infects plants and causes symptoms in different parts, including the stems, leaves, flowers, fruits, and roots; it is responsible for reduced plant quality and enormous yield losses worldwide [5,6]. Since it is a significant challenge to develop an effective and safe control method as well as that resistant cultivars are not available, farmers rely mainly on the application of fungicides to control Sclerotinia diseases and suffer from the disadvantage of fungicides [7,8]. Hence, there is an urgent demand for novel and secure methods to protect crops from this fungal disease.

Mycoviruses, also called fungal viruses, are present in various fungi. Most are latent and harmless to their hosts, although a few can bring exert significant effects on the morphology, reproduction, and virulence of their hosts [9,10]. The first successful case of mycovirus-based biocontrol involved European chestnut blight caused by *Cryphonectria parasitica* [11]. This caused mycoviruses to attract significant attention from phytopathologists as potential biological-control agents to manage fungal diseases. The fungus *S. sclerotiorum* is a fungal host with very diverse viruses, including hypovirulence-associated mycoviruses [12,13]. In particular, *Sclerotinia sclerotiorum* hypovirulence-associated DNA virus 1 converts its host from a necrotrophic pathogen to a mutualistic endophyte. A virus-infected strain could be explored as a plant vaccine to control rapeseed-stem rot and improve seed yield in fields, and the endophytic growth in rapeseeds could enhance the connection and strength of the microbial-interaction network in rapeseeds [14,15]. *Sclerotinia sclerotiorum* partitivirus 1 (SsPV1) was found to confer hypovirulence on its natural host, *S. sclerotiorum* strain WF-1, and SsPV1 was spread horizontally via hyphal contact to different vegetation-compatible groups of *S. sclerotiorum* and, interspecifically, to *S. nivalis* and *S. minor* [16]. *S. sclerotiorum* has complex vegetation-compatible groups, which limit the horizontal transmission of mycoviruses, but *Sclerotinia sclerotiorum* mycovirus 4 (SsMYRV4) can overcome this limitation and facilitate the transmission of other mycoviruses in *S. sclerotiorum* more effectively [17]. Therefore, investigating the mycoviruses in *S. sclerotiorum* might provide resources with which to control Sclerotinia disease in an eco-friendly manner.

The family *Alphaflexiviridae* belongs to the positive single-stranded RNA (+ssRNA)-virus group and is composed of five plant-infecting genera (*Allexivirus*, *Lolavirus*, *Mandarivirus Platypuvirus*, and *Potexvirus*,), two fungus-infecting genera (*Botrexvirus* and *Sclerodarnavirus*), and five unassigned species. Moreover, the genome of these viruses is monopartite-positive RNA, which is 5.9–9.0 kb in length and typically composed of five or six open reading frames (ORFs). The 5′-terminal ORF1 encodes a replicase with conserved methyltransferase (MTR), helicase (HEL), and RNA-dependent RNA polymerase (RdRp) domains, while the ORF closed to 3′-terminal encodes a coat protein (CP). The members of the family *Alphaflexiviridae* usually have flexuous filamentous particles (470 to 800 nm in length), and the majority of them have only mild effects on their hosts [18]. The first virus in the family *Alphaflexiviridae* reported in *S. sclerotiorum* was *Sclerotinia sclerotiorum* debilitation-associated RNA virus (SsDRV), from a hypovirulent strain Ep-1PN. This virus has only one ORF coding for RdRp, but lacks genes coding for coat protein and movement protein, making it distinct from all the reported members in the family *Alphaflexiviridae*. Thus, SsDRV was used to establish a genus *Sclerodarnavirus* as an exemplar [19].

In previous research, a virome-sequencing analysis of a set of *S. sclerotiorum* strains collected from diseased sunflowers and green beans in Washington State, USA, was carried out to discover novel mycoviruses (unpublished data). An assembled contig with a length of 6811 nt was found to encode a putative protein with a similarity to the replicase of Garlic virus C, a plant alphpaflexivirus; thus, was named *Sclerotinia sclerotiorum* alphaflexivirus 1 (SsAFV1). Through RT–PCR testing with specific primers of SsAFV1, two strains, AHS31 and GB862, were confirmed to harbor this virus. Furthermore, strain AHS31 also contains four other viruses. In this study, we cloned this alphaflexivirus from strain AHS31 and characterized its genomic features, virion morphology, and phylogenetic relationship to understand its biological properties in *S. sclerotiorum*.

## 2. Materials and Methods

### 2.1. S. sclerotiorum Isolates and Culture Conditions

The *S. sclerotiorum* strain, AHS31, was isolated from a sclerotium collected from a diseased sunflower donated by Dr. Weidong Chen of Washington State University. Ep-1PNA367, a virus-free strain with strong virulence, was used as a control [19]. All strains were cultured on potato dextrose agar (PDA) at 20 °C and stored on PDA slants at 4 °C.

### 2.2. Total-RNA Extraction and RT–PCR

Strain AHS31 was incubated on PDA plates covered with cellophane at 20 °C for 36–48 h. Total RNA was extracted from mycelia using TRIzol reagent (Invitrogen, Carlsbad, CA, USA), following the manufacturer’s instructions, and treated with DNase I to remove DNA contaminations. To verify the viruses in strain AHS31, cDNAs were synthesized by using a reverse-transcription kit (Transgen, Beijing, China) with an oligo(dT) primer. PCR amplification was performed with primers specific to each of the 5 viruses. The virus-free strain, Ep-1PNA367, was used as a negative control. The primers are listed in Table 1.

### 2.3. Terminal-Sequence Cloning of SsAFV1

Based on the 6811-nucleotide sequence obtained in the virome-sequencing data, the 3′ and 5′ terminal sequences of SsAFV1 were cloned through sequence-independent cDNA amplification and sequencing, as described previously [20]. Total RNA was extracted from AHS31 strain and used as a template for cDNA synthesis. An anchor-primer PC3-T7 loop (5′-p-GGATCCCGGGAATTCGGTAATACGACTCACTATATTTTTATAGTGAGTCGTATTA-OH-3′) was ligated to total RNA of strain AHS31 using T4 RNA ligase and the ligated RNA was extracted for reverse-transcription (RT) PCR to synthesize cDNA products. Primer PC2 complementary to the anchor primer and viral primers designed based on the available proximal-region sequences of SsAFV1 were used for the terminal amplifications. After purification by a gel-extraction kit (Omega Bio, Norcross, GA, USA), the final products were cloned into the pMD18-T vector (TaKaRa, Dalian, China) for sequencing. This step was repeated on three independent occasions to avoid laboratory interference. Primers used in this study are listed in Table 1.

### 2.4. Structural and Phylogenetic Analyses

The genomic structure of SsAFV1 was analyzed by DNAMAN and the putative ORFs and conserved domains or motifs were predicted through the ORF finder on NCBI website (http://www.ncbi.nlm.nih.gov, accessed on 5 July 2022) and in MOTIF research (https://www.genome.jp/tools/motif/, accessed on 5 July 2022). The genomic sequences of previously reported alphaflexiviruses referenced in this paper were retrieved from the NCBI GenBank database (http://www.ncbi.nlm.nih.gov/genomes, accessed on 4 July 2022). The percentage-identity matrix among the full-length replicases of selected alphaflexiviruses was generated through Clustal Omega 2.1, as described previously [21], and the image was processed by a custom R script. Multiple-sequence alignments were computed by MAFFT (version 7.427). The best-fitting amino-acid-substitution models were calculated using ModelFinder [22]. Phylogenetic analyses were performed using IQ-TREE (version 1.6) by the maximum likelihood (ML) method with 1000 bootstrap replicates.

### 2.5. Viral-Particle Purification and Observation of SsAFV1

Strain AHS31 was incubated on PDA plates at 20 °C for 5 days, after which mycelial mass (about 50 g, wet weight) was collected for virion extraction according to the method described previously by Xiao et al. [16], with minor modifications. Mycelial mass was homogenized in a juicer with 400 mL of buffer A (0.1 M sodium phosphate of pH 7.0), mixed with 0.1% (*w*/*v*) β-mercaptoethanol and shaken on ice for 60–90 min, followed by clarification with chloroform. The supernatant of virus fraction was collected by ultracentrifugation at 30,000 rpm with SW 32 Ti rotor (Beckman Coulter Inc., Brea, CA, USA) for 3 h and then subjected to sucrose-density gradient (15%, 30%, 45%, and 60%) ultracentrifugation at 30,000 rpm for another 3–4 h. Four separate fractions were further centrifuged at 30,000 rpm for 3 h and precipitates of every fraction were collected to examine SsAFV1. The SsAFV1 particles in the 30% sucrose layer were resuspended in 200 μL of buffer B (0.05 M sodium phosphate of pH 7.0). The final viral suspension was negatively stained with 2% (*w*/*v*) phosphotungstic acid solution (pH 7.4) and examined under a transmission-electron microscope (TEM; Model Tecnai G2, 200 kV, FEI Company, Hillsboro, OR, USA).

### 2.6. Biological Characteristics of AHS31 Strain

The colony morphologies of AHS31 and Ep-1PNA367 strains were observed at 7 days post-inoculation (dpi) on PDA. To assess the growth rate of these strains, the colony diameters were recorded at 24 and 36 h post-inoculation (hpi) at 20 °C. To evaluate the pathogenicity, detached leaves of rapeseed were inoculated with actively growing mycelial agar plugs (5 mm in diameter) of strains AHS31 and Ep-1PNA367, after which they were placed in an incubator at 20 °C with 90% relative humidity. Diseased lesions were measured and photographed at 36 hpi. Each treatment involved three replicates and was conducted at least twice.

### 2.7. Statistical Analyses

Growth rate and virulence assay data of *S. sclerotiorum* strains were subjected to analysis of variance in SAS (SAS Institute, version 8.1) and treatment means were compared using the least-significant-difference test at a significance level of a *p*-value of 0.01. Error bars were set according to standard deviation (SD) from sample means.

## 3. Results

### 3.1. Biological Characteristics of AHS31 Strain

**Figure 1:**
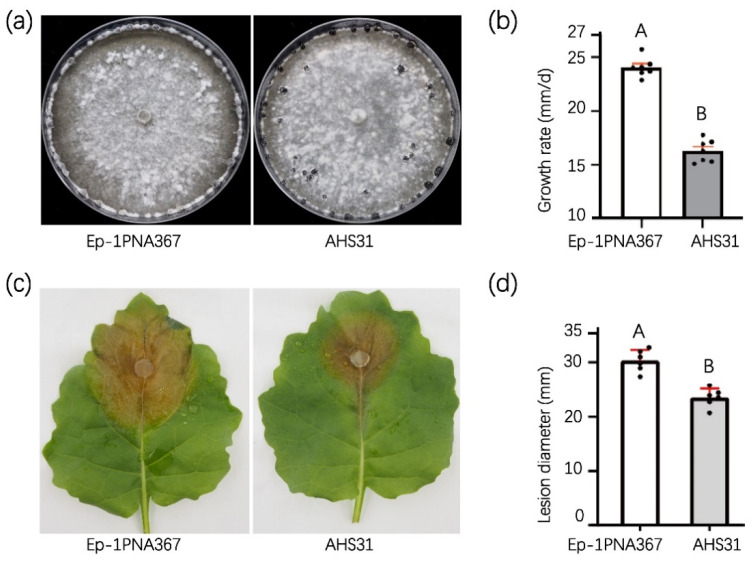
Biological characteristics of strains Ep-1PNA367 and AHS31. (**a**) Colony morphology of strains Ep-1PNA367 and AHS31. All the strains were cultured on PDA plates at 20 °C for 7 days. (**b**) Average growth rates of Ep-1PNA367 and AHS31 strains. (**c**,**d**) The pathogenicity assay of strain AHS31 (36 hpi). Error bars indicate standard deviation (SD) from sample means. Different uppercase letters on the top of each column indicate significant differences (*p* < 0.01).

Strain AHS31 exhibited a similar colony morphology to the control strain, Ep-1PNA367, but the sclerotia of theAHS31 strain matured faster than those of strain Ep-1PNA367 (Figure 1a). The growth rate of strain AHS31 (16.3 mm/d) was significantly lower than that of strain Ep-1PNA367 (24.0 mm/d) (Figure 1b). Furthermore, strain AHS31 presented lower virulence on the detached rapeseed leaves (Figure 1c) and produced lesions with an average diameter of 23.5 mm, which was significantly smaller than that of strain Ep-1PNA367 (30.3 cm, 36 hpi) (Figure 1d). These results demonstrate that AHS31 is a hypovirulent strain.

### 3.2. Virus Verification and Virion Observation

**Figure 2:**
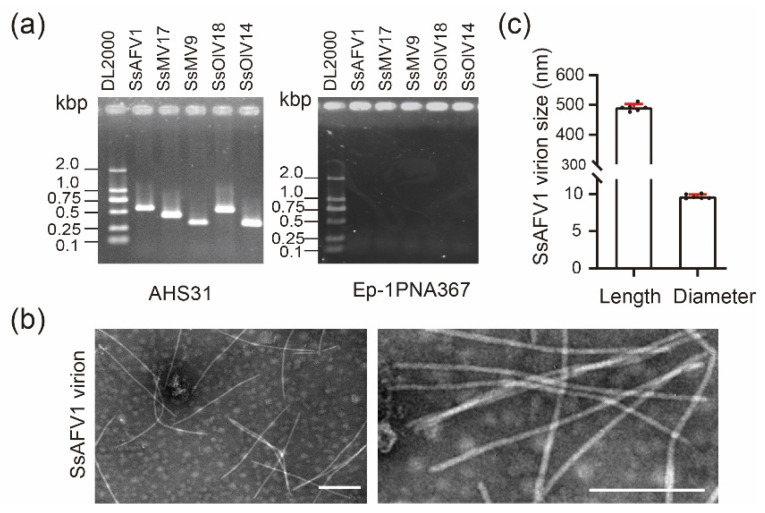
The viruses that infected the *S. sclerotiorum* strain, AHS31, and virions of SsAFV1. (**a**) Confirmation of the presence of five positive RNA viruses in strain AHS31 by RT–PCR. In control sets, ddH_2_O was used instead of RT products. Primers specific to each virus are listed in Table 1. (**b**) Virions of SsAFV1 observed under a transmission-electron microscope (TEM) after negative staining. The bar is 200 nm. (**c**) A histogram of SsAFV1 particle length and diameter. Error bars indicate standard deviation (SD) from sample means.

Previously, through high-throughput sequencing and data analysis, an assembled contig related to the plant virus GVC was found in strain AHS31. After RT–PCR verification, the results showed that a total of five positive single-stranded RNA (+ssRNA) viruses were detected in strain AHS31, including four established viruses, *Sclerotinia sclerotiorum* ourmia-like virus 14 (SsOlV14), *Sclerotinia sclerotiorum* ourmia-like virus 18 (SsOlV18), *Sclerotinia sclerotiorum* mitovirus 9 (SsMV9), *Sclerotinia sclerotiorum* mitovirus 17 (SsMV17), and the novel mycovirus SsAFV1 (Figure 2a).

The viral particles isolated from strain AHS31 were negatively stained by phosphotungstic-acid solution and examined by a TEM. Flexuous filamentous particles 9–10 nm in diameter and 480–510 nm in length were observed (Figure 2b). Since ourmia-like viruses and mitoviruses have no coat-protein genes, which are vital to form virion structures, and the morphologies of these particles met the standards of virion of alphaflexiviruses. Thus, they were proposed as the virions of SsAFV1.

### 3.3. Genomic Sequence and Organization of SsAFV1

**Figure 3:**
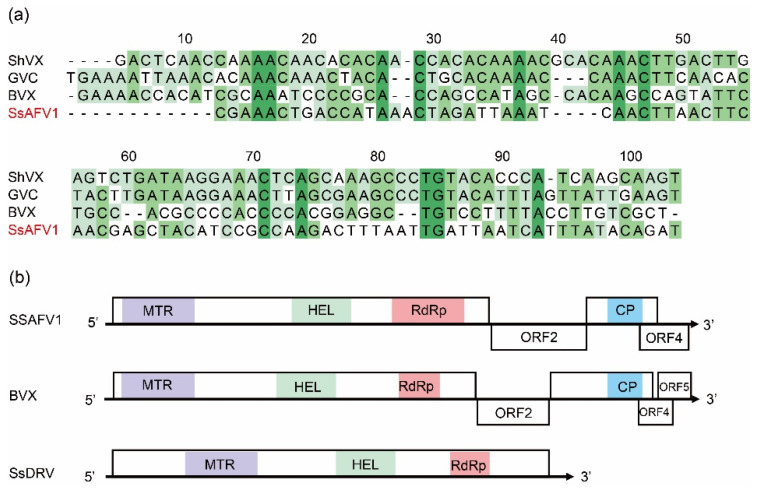
Genomic characteristics of SsAFV1. (**a**) Alignments of the nucleotide sequences of 5′-untranslated regions (UTRs) of SsAFV1 and selected viruses. (**b**) Schematic diagram of three fungal alphaflexivirus, SsAFV1, BVX, and SsDRV. Four conserved domains are shown as rectangular boxes in different colors. MTR, methyl transferase; HEL, RNA helicase; RdRp, RNA-dependent RNA polymerase; CP, coat protein.

The complete genome of SsAFV1 was obtained via sequence-independent amplification and sequencing and submitted to GenBank under the accession number ON993219. The genome of SsAFV1 comprised 6939 nucleotides (nt) with a tailing poly(A) in the 3′ terminus and a conservative 5′-UTR of 93 nt, including a 5′-CXXAA-3′ motif (Figure 3a). Additionally, its overall base compositions were 31.0% for A, 21.2% for T, 29.6% for C, and 18.2% for G. The SsAFV1 was predicted to have four putative ORFs. This is drastically different from all known plant alphaflexiviruses, which typically have five or six ORFs, as well as two representative fungal alphaflexiviruses, SsDRV (genus *Sclerodarnavirus*), with only a replicase gene, and Botrytis virus X (BVX, genus *Botrexvirus*) with five ORFs (Figure 3b).

The ORF1 was 4404 nt in length (nt position 94-4498) with a predicted molecular mass of 166.43 kDa and deduced to encode a viral RNA replicase of 1467 amino acid (aa) residues with an ordered array of the domains methyl transferase (MTR), RNA helicase (HEL), and RNA-dependent RNA polymerase (RdRp) (Figure 3b). The N-terminal MTR domain (PF01660) is positioned from 41 to 323 aa residue, which is the unique feature of the *Alphavirus* superfamily. The central viral-RNA-helicase domain (PF01443) ranging from 689 to 919 aa in residue, belonging to the SF1 helicase superfamily, contained an ATP binding AAA-like domain (PF12846) with a P-loop motif and a C-terminal domain with an AAA-like structural fold. The C-terminal RdRp domain (PF00978) ranging from 1086 to 1359 in aa residues, was vital for viral replication.

The ORF2 (nt position 4535–5645) encoded a 369 aa protein with a molecular mass of 41.52 kDa, sharing no identity with proteins of other viruses. The protein contained abundant serines, which is a typical characteristic of alphaflexiviruses [18], but its function remained unknown. The ORF3 (nt position 5687–6473), overlapping with ORF4, was predicted to encode a putative protein 187 aa in size and a molecular mass of 28.5 kDa. This protein shared an aa identity of more than 40% with the coat protein of Botrytis virus X (Figure 3b); thus, it was proposed as a coat protein of SsAFV1. Similarly, the ORF4 (nt position 6279–6843) in front of the poly (A) tail was predicted to encode a unique protein 187 aa in length. A conserved domain, DUF2457 (PF10446), was found ranging from 8 to 93 aa in ORF4, but its function was unknown. According to the Pfam database, this domain is mainly found in eukaryotes, but a few are present in viruses (Figure 3b).

### 3.4. Multiple-Alignment and Phylogenetic Analysis of Predicted Proteins of SsAFV1

**Figure 4:**
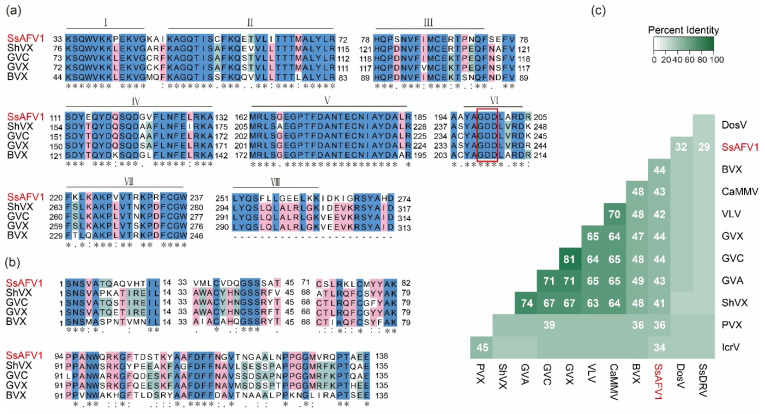
Multiple-alignment analysis of RdRp and CP of SsAFV1 and selected viruses. (**a**) Alignment of conserved RdRp of SsAFV1 and four selected alphaflexiviruses (Table S1). Eight conserved domains (I–VIII) among alphaflexiviruses are marked and GDD in the red rectangular box is the typical catalytic motif of positive single-RNA viral RdRp. Identical residues are indicated by asterisks and highlighted in blue. The conserved and semi-conserved amino-acid residues are indicated by colons and dots and highlighted in pink and green, respectively. (**b**) Alignment of conserved CP motifs of SsAFV1 and four selected alphaflexiviruses. Abbreviations of selected viruses are given in Table S1. (**c**) A percentage-identity matrix of viral replicase multiple-aligned via Clustal Omega 2.1 and generated by a custom R script. Abbreviations of viral names and GenBank accession numbers are listed in Table S1.

A multiple-sequence-alignment analysis was carried out using ClustalX 3.0 based on the core RdRp domain of SsAFV1, three members of *Allexivirus,* and a botrexvirus whose complete-nucleotide sequence is remarkably similar to that of SsAFV1. The results revealed that SsAFV1 has eight conserved motifs (I-Ⅷ) in the RdRp domain of ORF1, including a “GDD”, the typical signature of +ssRNA viral RdRp in motif Ⅵ (Figure 4a). Moreover, multiple-alignment analyses of the coat proteins showed conserved motifs among these chosen viruses, which might be vital to regulating the morphology of virus particles for members of the family *Alphaflexiviridae* (Figure 4b).

A percentage-identity matrix was constructed to observe the protein similarity among the replicases of selected viruses from the family *Alphaflexiviridae*. Interestingly, the results revealed that the RNA replicase of the SsAFV1 showed a similarity of 41–44% with those of the botrexvirus (Botrytis Virus X) and allexviruses, whereas it shared only 29% of identity with that of the SsDRV (Figure 4c).

**Figure 5:**
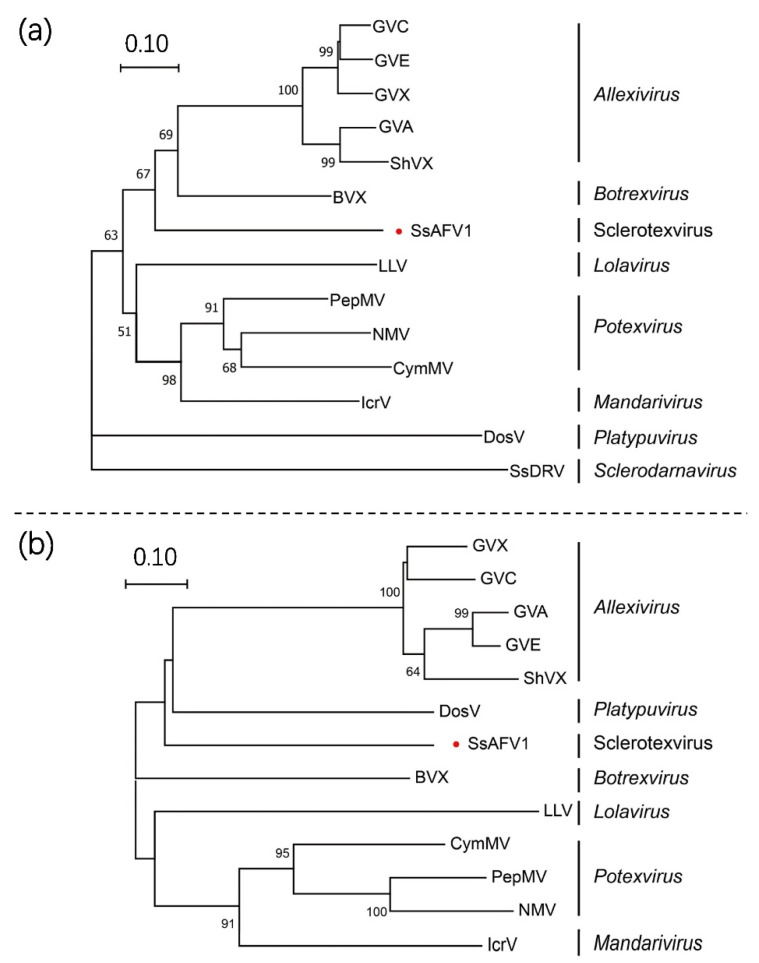
Phylogenetic analysis of SsAFV1 and representative members of the family *Alphaflexiviridae* based on the alignment of replicase (**a**) and CP (**b**). These ML trees were constructed as described with calculated best-fit model LG + I + G4 for replicase and LG + G4 for CP. The parameter of bootstrap was set as 1000 replicates and bootstrap values over 50% were indicated on branches. The virus studied in this paper was tagged with a red dot; the scale bar at the top left corresponds to the genetic distance. Details of these alphaflexiviruses are given in Table S1.

To confirm the evolutionary status of SsAFV1 in the family *Alphaflexiviridae*, a phylogenetic analysis was performed through the maximum likelihood (ML) method based on predicted protein sequences of replicase and CP of SsAFV1 and 13 representative viruses in seven genera of the family *Alphaflexiviridae*. Only 12 sequences were used for CP phylogenetic analysis due to the lack of coat protein in SsDRV. The phylograms of the replicase demonstrated that SsAFV1, *Allexivirus*, and *Botrexvirus* grouped into one clade, while SsAFV1 itself formed an independent branch (Figure 5a). This evolutionary relationship between SsAFV1 and the three aforementioned genera was in accordance with the replicase-matrix results described above. In addition, as shown in the CP phylogram exhibited in Figure 5b, SsAFV1 and the other tree genera (*Allexivirus, Lolacirus*, and *Botrexvirus*) gathered in a sub-clade and simultaneously formed a separate branch in the phylogenetic tree.

## 4. Discussion

Here, we characterized a novel mycovirus, SsAFV1, from a hypovirulent strain, AHS31, of the plant pathogenic fungus *S. sclerotiorum*. Based on the genomic structure, virion morphology, and evolutionary relationship, SsAFV1 is a new member of the family *Alphaflexiviridae*. The SsAFV1 displays typical characteristics of alphaflexiviriuses in its genomic architecture, especially the invariant array of three conserved motifs in the replicase and eight conserved domains in the RdRp; in addition, the last two ORFs overlapped by 195 nt, which is the case in the majority of alphaflexiviruses. The virion of SsAFV1 was similar to those of typical alphaflexiviriuses, but it was much shorter than that of a fungus-infecting alphaflexiviruse (botrevirus) [18]. Regarding the phylogenetic relationship, it should be noted that SsAFV1 and BVX are very close to plant alphaflexiviriuses. Although SsAFV1 shares the same host as SsDRV, they have a distant evolutionary relationship and low nucleotide-sequence identity. These results suggest that SsAFV1 is independent of viruses in all established genera and may represent a novel genus. Thus, a new genus, Sclerotexvirus, is proposed to accommodate this novel mycovirus in the family *Alphaflexiviridae*.

According to the BLASTp search of the NCBI protein database, the replicase of SsAFV1 was the closest to the members of the genus *Allexivirus*, displaying aa identities of 44% with GVC and GVX. The high degree of similarity suggested that they might share a common ancestry. Two hypotheses for the origin of mycoviruses were discussed in previous research. The first is that they are of an unknown but ancient origin and have co-evolved along with their hosts. The second is that they moved from a fungal-plant host to the fungus relatively recently [23]. Therefore, it is possible that SsAFV1 originally evolved from a plant virus or that, together with plant alphaflexiviruses, its ancestry is unknown. In laboratory conditions, several plant viruses, cucumber mosaic virus (CMV), Brome mosaic virus (BMV), and tobacco mosaic virus (TMV) were reported to exist and replicate in various fungi [24,25,26], even in an oomycete [27]. Furthermore, shreds of evidence from recent research proved that a phytopathogenic fungus *Rhizoctonia solani* is naturally infected by a plant virus, CMV [28]. A similar case occurred in a dsRNA virus of the family *Partitiviridae* that partitiviruses occasionally exchanged between fungal and plant hosts, possibly involving endophytic fungi in particular [29]. Thus, the horizontal transfer between plants and fungi in nature helps these viruses to master additional evolutionary pathways to generate novel viruses with higher adaptability and survivability.

Moreover, plant alphaflexiviruses usually have five or more ORFs, but the number of ORFs appeared to vary among fungal alphaflexiviruses. Botrexvirus contains five ORFs, in accordance with the standard of plant-infecting genera of *Alphaflexiviridae*, while sclerodarnavirus comprises only a single ORF, making it a unique case in this family [30]. Till now, SsAFV1 is the first alphaflexivirus containing four ORFs. The decrease in the number of ORFs was probably due to deletions during the transmission and evolution process. Generally, RNA genomes were prone to errors within the replication process, but it was an evolutionary advantage to generate sequence variants for better adaption to new environments [31,32,33]. Simple genomic structures allow highly efficient viral replication; therefore, it is likely that mycoviruses to cast off redundant and unnecessary segments within the biological process.

In nature, a single fungal strain often harbors multiple mycoviruses. Examples occurred frequently in *S. sclerotiorum* [19], *Magnaporthe oryzae* [34], *Botrytis cinerea* [35], and *Rosellinia necatrix* [36]. Many hypovirulent *S. sclerotiorum* strains were found to be infected by more than one mycovirus [16,37,38]. As shown in the present study, besides SsAFV1, strain AHS31 also accommodates two mitoviruses and two ourmia-like viruses. The SsAFV1 was also detected in another *S. sclerotiorum* strain, GB862 (MW454906), with strong virulence, and the genome of SsAFV1/GB862 is 6902 bp in length. These two isolates of SsAFV1 share a nucleotide-sequence identity of 97%; that is to say, they are independent isolates of the same virus in different *S. sclerotiorum* strains. Furthermore, mitoviruses and ourmia-like viruses in fungi generally infect latently and exert only a slight influence on their hosts [39,40]. Thus, it is likely that the co-infection of all the mycoviruses confers hypovirulence on strain AHS31. However, the underlying mechanisms are unknown and should be investigated in the future.

In summary, we reported a novel mycovirus, SsAFV1, in the family *Alphaflexiviridae* in a hypovirulent *S. sclerotiorum* strain. The SsAFV1 mycovirus contained four ORFs and its virion was flexuous filamentous. Based on the analysis of the viral genome and phylogenetic relationship, a new genus, Sclerotexvirus, was proposed to accommodate SsAFV1.

## Figures and Tables

**Table 1:** Primers involved in this study.

Primer	Sequence (5′ to 3′)	Length (bp)	Description
**AFV1F**	ATAGGTTCGCTCAGCCTTTTG	21	For SsAFV1 detection
**AFV1R**	CAGCCCTCTACACCGCATT	19	
**MV9F**	TATCAGGATTCATACCGAGGCA	22	For SsMV9 detection
**MV9R**	CACCGACAAAGGAAAGAAGGAG	22	
**MV17F**	AGCAGAGTGGACCAGGCTATT	21	For SsMV17 detection
**MV17R**	TGTTCACCCTATCCCATCATTT	22	
**OlV18F**	TGTGACGGCTGAGAAGTTGAA	21	For SsOlV18 detection
**OlV18R**	TCCCATCCTCGTTGTCTGAAT	21	
**OlV14F**	GGAAGACGGCAGCAGCAAA	19	For SsOlV14 detection
**OlV14R**	TCGCCACTCCCAGAAAAGC	19	
**PC2**	CCGAATTCCCGGGATCC	17	For nested PCR
**3AFV1L**	CACTCAACGCTCACTTGCTC	20	For 3′ terminal-sequence cloning of SsAFV1
**3AFV1S**	GAACGACACCACCACAATGG	20	
**5AFV1L**	GACCAGGGGATGTTCGCATA	20	For 5′ terminal sequence cloning of SsAFV1
**5AFV1S**	GCGTGTGAGTGTAATTGCGT	20	

## Data Availability

Not applicable.

## Supplementary Materials

The following supporting information can be downloaded at: https://www.mdpi.com/article/10.3390/v15020339/s1. Table S1: Details of viruses referred to in this study [41,42,43,44,45,46,47,48,49,50,51,52,53,54,55,56].
