# Supplementary material for: Characterization of a Fungal Virus Representing a Novel Genus in the Family Alphaflexiviridae
Source: Viruses. 2023 Jan 25;15(2):339. doi: 10.3390/v15020339 (PMC9967154; doi:10.3390/v15020339)
Supplement: Supplementary file 1 [file viruses-15-00339-s001.zip › viruses-2126814-supplementary.pdf]

viruses

MDPI

Table S1. Details of viruses referred to in this study.

Name	Abbreviation	Accession	Length (nt)	References
Cymbidium mosaic virus	CymMV	NC_001812.1	6227	[41]
Cassia mild mosaic virus	CaMMV	MN031278.1	7829	[42]
Pepino mosaic virus	PepMV	NC_004067.1	6410	[43]
Narcissus mosaic virus	NMV	NC_001441.1	6955	[44]
Indian citrus ringspot virus	IcrV	NC_003093.1	7560	[45]
Lolium latent virus	LLV	NC_010434.1	7674	[46]
Vanilla latent virus	VLV	NC_035204.1	7462	[47]
Strawberry mild yellow edge virus	SMYEV	NC_003794.1	5966	[48]
Donkey orchid symptomless virus	DosV	NC_022894.1	7838	[49]
Botrytis virus X	BVX	NC_005132.1	6966	[35]
Garlic virus A	GVA	NC_003375.1	8660	[50]
Garlic virus C	GVC	NC_003376.1	8405	[50]
Garlic virus X	GVX	NC_001800.1	8106	[51]
Garlic virus E	GVE	NC_004012.1	8451	[52]
Shallot virus X	ShVX	NC_003795.1	8832	[53]
Potato virus X	PVX	NC_011620.1	6435	[54]
Sclerotinia sclerotiorum mitovirus 9	SsMV9	KF913884.1 [a]	2562	[55]
Sclerotinia sclerotiorum mitovirus 17	SsMV17	KP900924.1 [a]	2447	[55]
Sclerotinia sclerotiorum debilitation-associated RNA virus	SsDRV	NC_007415.1	5470	[19]
Sclerotinia sclerotiorum ourmia-like virus 18	SsOlV18	MW454909.1 [a]	3336	Unpublished
Sclerotinia sclerotiorum ourmia-like virus 14	SsOlV14	MT646410.1 [b]	3368	[56]

[a] complete coding sequence (CDS); [b] partial sequence.
